# Roar of a Champion: Loudness and Voice Pitch Predict Perceived Fighting Ability but Not Success in MMA Fighters

**DOI:** 10.3389/fpsyg.2019.00859

**Published:** 2019-04-30

**Authors:** Pavel Šebesta, Vít Třebický, Jitka Fialová, Jan Havlíček

**Affiliations:** ^1^National Institute of Mental Health, Klecany, Czechia; ^2^Faculty of Humanities, Charles University, Prague, Czechia; ^3^Faculty of Science, Charles University, Prague, Czechia

**Keywords:** speech, roar, vocalization, handgrip, competition, perception, human

## Abstract

Historically, antagonistic interactions have been a crucial determinant of access to various fitness-affecting resources. In many vertebrate species, information about relative fighting ability is conveyed, among other things, by vocalization. Previous research found that men's upper-body strength can be assessed from voice. In the present study, we tested formidability perception of intimidating vocalization (roars) and a short utterance produced by amateur male MMA fighters attending the amateur European Championships in relation to their physical fitness indicators and fighting success. We also tested acoustic predictors of the perceived formidability. We found that body height, weight, and physical fitness failed to predict perceived formidability either from speech or from the roars. Similarly, there was no significant association between formidability of the roars and utterances and actual fighting success. Perceived formidability was predicted mainly by roars' and utterances' intensity and roars' harmonics-to-noise ratio and duration. Interestingly, fundamental frequency (F_0_) predicted formidability ratings in both roars and utterances but in an opposite manner, so that low F_0_ utterances but high F_0_ roars were rated as more formidable. Our results suggest that formidability perception is primarily driven by intensity and duration of the vocalizations.

## Introduction

Historical and ethnographic evidence shows that physical encounters were a frequent way of resolving conflicts (Manson et al., 1991; Keeley, 1997). Cross-culturally, man's fighting ability is a powerful determinant of access to resources (Daly and Wilson, 1988). These findings are complemented by psychological studies which show that stronger men are more prone to anger (Archer and Thanzami, 2007; Sell et al., 2009b). One may therefore expect that cognitive processes evolved for assessing the threat potential of a prospective opponent (Sell et al., 2009a; Puts, 2010). Earlier research tended to focus on visual cues to the threat potential. It has been demonstrated, for instance, that people can relatively accurately assess physical strength from images of body and face (Sell et al., 2009a; Holzleitner and Perrett, 2016; Kordsmeyer et al., 2018). Moreover, it seems that based on facial images raters can predict winners of mixed martial arts (MMA) fights (Třebický et al., 2013; Little et al., 2015; but see Třebický et al., 2019).

The cues to threat potential are not restricted to the visual modality but evidence regarding vocal indicators of threat potential is rather mixed. On one hand, it was reported that both men and women can accurately assess men's physical strength from voice irrespectively of the language used (Sell et al., 2010). On the other hand, fighting ability assessed by acquaintances did not correlate with ratings of fighting ability based on vocal stimuli (Doll et al., 2014). Han et al. (2017) likewise reported no association between a composite measure of threat potential, consisting of handgrip strength, body height and weight, and the perceived vocal threat potential.

Importantly, all of the abovementioned studies used speech as their acoustic stimuli. Humans, however, produce also various other vocalizations, such as laughter, roars, screams and grunts, and these so far received only limited attention. This contrasts with evidence from a number of vertebrate species, including primates, which shows that vocal displays are frequently part of male intrasexual competition (Bradbury and Vehrencamp, 2011) and can indicate fighting ability (for evidence in red deer, see Clutton-Brock and Albon, 1979; for baboons, see Kitchen et al., 2003). In humans, it has recently been shown that tennis players who produce grunts with a lower fundamental frequency (F_0_) are more likely to win and listeners can to some extent predict match outcome from the grunts (Raine et al., 2017). Similarly, Raine et al. (2018a) reported that listeners accurately assess relative strength and body height from aggressive roars in both men and women.

In our complementary study, we tested predictors of perceived formidability using acoustic cues. It ought to be noted, however, that Raine et al. (2018a) and the current study differ in several important respects. First of all, Raine et al. focused on two important components of threat potential (height and strength), but threat potential and/or *perceived* formidability undoubtedly include other components as well. These may include morphological characteristics, such as body weight and lean muscle mass, as well as physical abilities other than isometric strength, for instance respiratory fitness. Secondly, while one can expect that threat potential is a predictor of outcomes of real-life fights, it cannot be entirely equated with fighting success.

To address these questions, we recorded both verbal and non-verbal vocalizations (utterances and roars) of amateur male MMA athletes along with (i) measurements of their body composition, isometric strength, and spirometry, and collected data regarding their (ii) fighting success.

We hypothesized that formidability perceived from vocalization should correlate with height, weight, and muscle mass as well as physical fitness indicators, such as strength and lung capacity. We also predicted that perceived formidability is positively associated with fighting success. Further, we performed an acoustic analysis to identify which parameters predict the perception of formidability from both roars and utterances. We hypothesized that perceived formidability is related to the F_0_ and intensity in both verbal and non-verbal vocalizations.

## Materials and Methods

All procedures applied in this study were in accordance with ethical standards of the responsible committee on human experimentation and with the Helsinki Declaration. The study was approved by the Institutional Review Board of the National Institute of Mental Health, Czech Republic (Ref. num. 28/15). All target participants were provided with a brief description of the study and approved their participation by signing informed consent. The present study is part of a larger project investigating multi-modal perception of traits associated with sexual selection and characteristics related to competition outcome.

### Targets

Data collection took place during 2016 IMMAF European Open Championships of Amateur MMA held in Prague (Czech Republic), which hosted a total of 155 contestants (incl. 20 women) from 30 countries (based on data from MyNextMatch.com). Contestants were approached by researchers during registration on site, 1 day before the start of the tournament. We focused on male athletes because championship attendance was highly biased toward male athletes and we thus managed to collect data from only three female athletes.

Forty male amateur MMA fighters (mean age = 24, *SD* = 4.4, range = 19–33 years), naïve to our project's aims, participated in the study. To assess a possible effect of weight category, we merged the weight categories used by competition organizers (Flyweight, *N* = 1; Bantamweight, *N* = 7; Featherweight, *N* = 4; Lightweight, *N* = 4; Welterweight, *N* = 7; Middleweight, *N* = 5; Light Heavyweight, *N* = 5; Heavyweight, *N* = 4; and Super Heavyweight, *N* = 3) into just three categories: Lightweight (*N* = 12; consisting of Flyweight, Bantamweight, and Featherweight categories), Middleweight (*N* = 16; consisting of Lightweight, Welterweight, and Middleweight categories) and Heavyweight (*N* = 12; consisting of Light Heavyweight, Heavyweight, and Super Heavyweight) following procedure in Třebický et al. (2013). All targets reported their basic demographics, age, and total fighting record, from which computed their fighting success as a proportion of the number of wins relative to the total number of fights. Fighting success was calculated only for fighters whose record included more than two fights. Analyses involving fighting success are therefore based on 29 individuals. For technical reasons, we managed to obtain lung capacity measures from 34 individuals. For descriptive statistics, see Table 1. All other analyses are based on the complete dataset of 40 individuals. Participants in the study were financially reimbursed with 400 CZK (app. €15) and verbally debriefed upon completing their participation.

**Table 1:** Target descriptive statistics.

**Characteristic**	**N**	**Mean**	**SD**	**Minimum**	**Maximum**
Age (yrs)	40	24	4.397	19	33
Score (wins/fights ratio)	29	0.682	0.208	0	1
Body Height (cm)	40	176.7	8.687	161.2	196
Body Weight (kg)	40	79.345	20.593	56.5	134.1
Body Fat (kg)	40	8.047	9.077	1.7	40.1
Muscle Mass (kg)	40	67.775	12.254	50.3	97.6
Maximal Handgrip Strength (right) (kp)	40	52.225	10.34	34.2	76
Maximal Handgrip Strength (left) (kp)	40	51.625	9.127	34.3	68
Mean Handgrip Strength (kp)	40	49.243	8.939	33.283	63.817
FVC (l)	34	4.515	0.879	2.46	6.55
FEV_1_ (l)	34	4.296	0.747	2.45	5.77
PEF (l)	34	8.469	2.471	3.86	11.73
Roar F_0_ (Hz)	40	392.85	56.893	243.503	492.725
Roar F_3_ (Hz)	40	2624.125	233.835	2196	3150
Roar HNR (dB)	40	9.169	5.068	2.926	24.786
Roar Intensity (dB)^*^	40	54.599	4.68	44.619	61.819
Roar Duration (s)	40	0.641	0.312	0.138	1.280
Utterance F_0_ (Hz)	40	127.771	18.387	100.844	179.099
Utterance F_3_ (Hz)	40	2546.02	98.66	2310.286	2727.487
Utterance HNR (dB)	40	8.929	2.88	3.452	14.205
Utterance Intensity (dB)^*^	40	13.721	3.803	6.643	23.057
Utterance Duration (s)	40	8.024	2.81	2.898	19.41

* *Intensity values are reported as analyzed from the original recordings (i.e., before being post-processed)*.

### Body Measurements

Body height was measured by Vít Třebický using anthropometer Trystom A-213. Participants were standing with their back against a wall, looking directly ahead, and body height was measured from Vertex to ground to a nearest millimeter (Hall et al., 2007).

Body weight, amount of body fat, and muscle mass were measured by Jitka Fialová (JF) using bio impedance Tanita MC-980 scale (Athlete setting; Vaara et al., 2012). Testing was performed in a standing position while standing on and holding in hands the measuring electrodes with arms hanging freely along the body. Participants were wearing underwear only (Pinilla et al., 1992).

### Physical Fitness Measurements

Handgrip strength was measured by JF using Takei TKK 5401 digital hand dynamometer (Vidal Andreato et al., 2011; Bonitch-Góngora et al., 2013). While undergoing the handgrip test, the athletes were instructed to stand straight with arms alongside their body. They had 3 attempts with each hand, alternated hands between attempts, and we used the “best test” method, meaning the attempt with the highest value of handgrip strength for each hand was recorded. Maximal handgrip strength between left and right hand was closely correlated (*r* = 0.808 95% CI [0.664, 0.894], *p* < 0.001, *N* = 40) and paired sample *t*-test showed no statistical difference between the maximal strength of left and right hand [*t*_(39)_ = 0.618, *p* = 0.54, mean difference = 0.6 kp]. In all further analyses involving handgrip strength, we therefore represent handgrip strength by the mean of both hands “best test” score.

Measures of lung capacity were taken by JF using MicroLab ML3500 MK8. Three standing forced vital capacity (FVC) maneuvers were performed, “best test” method applied, and we recorded the maneuver with the highest recorded FVC value along with Forced expiratory volume in the first second (FEV_1_) and Peak expiratory flow (PEF). The “best test” method is a widely used and recommended approach in research employing spirometry (Crapo et al., 1981; Havryk et al., 2002). FVC is the maximal volume of air exhaled with maximally forced effort from maximal inspiration delivered during expiration made as forcefully and completely as possible. In other words, it is vital capacity performed with a maximally forced expiratory effort. FEV_1_ is the maximal volume of air exhaled in the first second of forced expiration from a position of full inspiration, and PEF represents the maximum expiratory flow achieved by maximum forced expiration from the point of maximal lung inflation (Miller et al., 2005).

### Vocal Stimuli Recordings and Processing

Acoustic stimuli were recorded by Pavel Šebesta (PŠ) using Sony PMC-D90 portable audio recorder (in-built microphone sensitivity 20–40 kHz). Recorder was equipped with a windscreen (AD-PCM1), mounted on a tripod with acoustic reflection shield and placed in a portable, acoustically treated booth to reduce any potential echoes and ambient noises. Recordings were captured at 24 bit/96 kHz in WAV format. Participants stood 1.5 meters from the recorder and Levels setting was kept constant in the course of all recordings to standardize recording intensity and to prevent clipping.

Participants were instructed to count from 1 to 10 in their native language and then perform three intimidating roars (their instruction was: “Roar three times, as much as you can, to intimidate a potential opponent”). For ratings and analyses, we use only the second roar because the first might be affected by the novelty of the task and the third by a potential decrease of effort (for differences between the three roars, see Supplementary Material Tables S1–S11). For examples of roars see Audios S1, S2 and for utterances see Audios S3, S4.

Subsequent processing and acoustical analysis were performed by PŠ in Audacity 2.1.3 (Audacity Team, 2018) and Praat 5.4.09 (Boersma and Weenink, 2015). Roars and utterance levels were increased by +20 dB and +35 dB, respectively, while interindividual variation in vocalization intensity remained unmodified. This intensity adjustment was necessary because most utterance recordings were not sufficiently loud even at the highest volume settings. The employed adjustments in roars was the highest possible that did not introduce clipping in any of the recordings. We measured the mean intensity and duration of volume-adjusted utterances and roars. Mean F_0_ was measured by autocorrelation method. Preset parameters for F_0_ extraction were used, with a 75 Hz pitch floor in accordance with Praat programmers' recommendations and 300 Hz pitch ceiling based on a visual inspection of spectrographs (for similar approach see also Šebesta et al., 2017). The 300 Hz pitch ceiling recommended for utterances was not suitable for the roars. We visually inspected Praat's pitch contours in the Editing window. Most roar recordings showed erroneous F_0_ measurements (see Figure S1 for an example), which rendered the standard Praat's F_0_ extraction method unreliable for this type of acoustic stimuli (for similar issues with F_0_ extraction, see Raine et al., 2017). F_0_ tracking frequently failed in the middle of recording or even unexpectedly “jumped down.” This is possibly due to chaotic and subharmonic phenomena found in roars (Fitch et al., 2002). For this reason, we decided to use, as a F_0_ analog, the long-term averaged Fast Fourier transformed (FFT) spectral peak frequency (see Figure S2 for an example), corresponding to the first harmonic (verified by a visual inspection of harmonic structure). Further, we used standard Praat methods for harmonics-to-noise ratio (HNR; autocorrelation method, preset parameters) measurements for whole utterance recordings, and one second long snips from the initial part of the roars close to the spectrogram plateau where Praat's autocorrelation algorithm was able to track F_0_. Mean formant levels in speech (F_1_–F_4_) were measured by Burg method. In roars, however, only a peak around 2–3 kHz (which is in expected range for the third formant) was apparent by a visual inspection of long-term average spectrums (LTAS) and clearly distinguishable from other harmonics. Audacity's “Plot spectrum” feature (Spectrum, 1,024 window size, Hanning window) was used for the 2–3 kHz peak measurement. Because we were able to reliably extract only the third formant (F_3_) from roars and the first and second formants in speech are highly affected by speech content, we decided to use in subsequent analyses only the third formant of both utterances and roars to enable comparison.

### Rating Sessions

In total, 31 men (mean age = 27.1, *SD* = 5.2, range = 20–36 years) and 32 women (mean age = 24.4, *SD* = 4.3, range = 18–33 years), mainly students at the Charles University, Prague, Czech Republic, took part in rating sessions.

Raters were recruited via social media advertisements and mailing lists of participants from previous studies. After completing participation, they were financially reimbursed with 100 CZK (~ €4), a small snack, and received a debriefing leaflet about the purpose of the study.

Raters were asked to assess the formidability (“Jak moc by byl tento muž úspěšný, kdyby se dostal do fyzického souboje?”/“How successful would this man be if he was involved in a physical confrontation?”) of a given recording on a 7-point verbally anchored scale (from “1–velice neúspěšný”/“not successful at all,” to “7–velice úspěšný”/“highly successful”). Each participant rated all roar and utterance stimuli. To reduce participant fatigue, the rating was divided in two sessions 1 week apart. In the first session, participants rated half of the set of all roars and utterances in a randomized order. Individual stimuli within the set were randomized as well. In the second session, participants rated the remaining half of the stimuli in the same fashion.

Ratings took place in a quiet perception lab room with negligible ambient sounds. Focusrite Scarlett Solo Gen 2 audio I/O interface (22 Hz−22 kHz RCA output) and two Yamaha HS-7 active reference studio monitor speakers (43 Hz−30 kHz @ 95W, LF 60 W, HF 35 W output) were used to present stimuli in WAV format. Raters were seated 2.8 meters in front of and in focus of the speakers. We opted for speakers, instead of commonly used headphones, because it is a more ecologically valid approach to presenting stimuli in terms of sonic characteristics of roaring. Loudness of the playback was kept standard during the presentation, with the loudest roar registering 87 dB (measured with OnePlus One smartphone and Smart Tools® Sound meter 1.6.12 app). This is a level which, all authors agreed, was very naturalistic but not overwhelmingly loud.

### Statistical Analyses

All statistical tests were performed in JASP 0.9.0.1 (JASP Team, 2018) and jamovi 0.9.1.7 (jamovi project, 2018). McDonald's ω statistics was used to estimate interrater agreement (Dunn et al., 2014). To test for potential sex differences in ratings, a paired samples *t*-test was carried out. Associations between ratings by men and women were tested by bivariate correlations using Pearson's r coefficient with 95% CIs [lower limit, upper limit]. Potential differences between the maximal strength of left and right hand were tested with paired samples *t*-test, and associations between the left and right hand strength were tested by bivariate correlations using Pearson's r coefficient with 95% CIs. Cohen's d, as an effect size measure, was used for means comparisons. To assess the relative contribution of performance-related and acoustic measures to the perceived roar and utterance formidability, we performed Linear mixed effects model (using REML fit) with individual rater ID and target stimuli ID as random intercepts. This approach accounted for variation on the level of individual raters and for variation on the level of individual stimuli. It also accounted for potential bias due to the data aggregation. To assess acoustic predictors of fighting success, we ran a linear regression analysis (Enter method). As measures of variability explained by regression, we list model R^2^ values, while standardized βs and their 95% CI are reported for entered coefficients.

### Data Availability

Datasets generated and analyzed during the current study are available in the Supplementary Material of this article (Tables S20, S21).

## Results

### Sex Differences in Perceived Formidability

#### Utterances

McDonald's ω scores of male (ω = 0.954) and female (ω = 0.933) ratings of formidability of utterances showed a high interrater agreement. We have therefore used mean formidability ratings given to the individual utterances separately by male and female raters. Perceived formidability of utterances was likewise highly correlated between men and women (*r* = 0.93 95% CI [0.871, 0.963], *p* < 0.001, *N* = 40). Paired sample *t*-test showed a statistically significant sex difference in formidability ratings with men giving higher ratings [*t*_(39)_ = 9.165, *p* < 0.001, Cohen's *d* = 1.449, mean difference = 0.368] (for descriptive statistics, see Table 2). Although mean ratings of utterance formidability differed between sexes, all further analyses are reported with ratings combined because the results are virtually the same when analyzed separately. For results based on female and male ratings separately, see Supplementary Material Tables S12–S19.

**Table 2:** Formidability rating descriptive statistics.

**Stimuli**	**Raters**	**Rating**		**McDonald's ω**
		**Mean**	**SD**	
Utterance	Total	3.404	0.663	0.947
	Women	3.22	0.687	0.933
	Men	3.588	0.664	0.954
Roar	Total	3.92	1.15	0.939
	Women	3.986	1.237	0.924
	Men	3.854	1.079	0.953

#### Roars

McDonald's ω scores of males (ω = 0.953) and females (ω = 0.924) ratings of roar formidability showed a high interrater agreement. In subsequent analyses, we have therefore used mean formidability ratings given to the individual roars separately by male and female raters. Further, we found a high correlation between roar formidability ratings assigned by men and by women (*r* = 0.973 95% CI [0.95, 0.986], *p* < 0.001, *N* = 40). Paired sample *t*-test showed statistically significant difference between the sexes in roar formidability ratings with women giving higher ratings [*t*_(39)_ = 2.695, *p* = 0.645, Cohen's *d* = 0.426, mean difference = 0.132]. For descriptive statistics, see Table 2.

### Formidability of Utterances and Roars as a Predictor of Fighting Success

To test whether formidability perception from roars and utterances predicts fighting success, we ran bivariate Pearson's correlations. We found that neither in utterances (*r* = −0.045 95% CI [−0.405, 0.327], *p* = 0.817, *N* = 29) nor in roars (*r* = −0.115 95% CI [−0.462, 0.263], *p* = 0.554, *N* = 29) was formidability perception associated with actual fighting success. To explore whether the effect is modulated by the weight categories, we grouped the fighters in three weight categories (lightweight, middleweight, and heavyweight) and entered this variable into the linear regression. Even after this modification, however, the overall model was not formally significant either in utterances [*F*_(3, 25)_ = 1.841, *p* = 0.166, R^2^ = 0.181] or in roars [*F*_(3, 25)_ = 0.683, *p* = 0.571, R^2^ = 0.076].

### Physical Fitness Predictors of Perceived Formidability

First, were ran exploratory correlational analyses to assess relationships between the physical fitness variables (see Supplementary Material Table S22). Body weight, muscle mass, and fat mass were all highly positively intercorrelated (rs > 0.757, ps < 0.001, *N* = 40). To avoid collinearity and to facilitate interpretation of the findings, we used only body weight in the subsequent analyses. FVC and FEV_1_ spirometry measures were likewise highly positively correlated (*r* = 0.935 95% CI [0.872, 0.967], *p* < 0.001, *N* = 34), which is why we decided to omit the FEV_1_ from subsequent analyses.

Linear mixed model analyses were run with age, height, weight, FVC, PEF, and handgrip strength as fixed effect predictors to assess whether physical fitness parameters predict the perceived formidability of utterances and roars. The overall model for utterances explained 44.9% of variance (R^2^ conditional) and fixed factors explained 5.4% of variance (R^2^ marginal). None of the physical fitness predictors for the formidability of utterances was formally significant. The overall model for roars explained 60.1% of variance (R^2^ conditional), while fixed factors explained 8.2% of variance (R^2^ marginal). Similarly, none of the predictors of perceived formidability in roars were significant. For an overview of the results, see Table 3.

**Table 3:** Summary of linear mixed effects model analysis for physical fitness predictors of perceived fighting ability based on utterances and roars.

	**Predictors**	**Estimate**	***t***	***p***	**95% CI**
Utterances	Age (yrs)	0.024	0.923	0.364	−0.027, 0.074
	Height (cm)	0.050	1.943	0.062	−0.00004, 0.010
	Weight (kg)	−0.010	−1.039	0.308	−0.028, 0.009
	FVC (l)	−0.277	−1.953	0.061	−0.556, 0.0001
	PEF (l)	0.016	0.323	0.749	−0.081, 0.113
	Handgrip strength (kp)	0.011	0.735	0.469	−0.019, 0.041
Roars	Age (yrs)	0.073	1.505	0.144	−0.022, 0.169
	Height (cm)	−0.044	−0.899	0.377	−0.138, 0.051
	Weight (kg)	0.017	0.971	0.340	−0.017, 0.052
	FVC (l)	0.402	1.496	0.146	−0.125, 0.929
	PEF (l)	−0.114	−1.223	0.232	−0.297, 0.069
	Handgrip strength (kp)	0.004	0.153	0.880	−0.052, 0.061

### Acoustic Predictors of Perceived Formidability

Linear mixed model analyses were run to predict perceived formidability from utterances and roars with F_0_, F_3_, HNR, intensity, and duration entered as independent predictors. For utterances, the overall model explained 44.1% of variance (R^2^ conditional), while fixed factors explained 9.6% of variance (R^2^ marginal). We found that F_0_ and intensity are significant predictors of perceived formidability. In the case of roars, the overall model explained 57% of variance (R^2^ conditional) and fixed factors explained 37.5% of variance (R^2^ marginal). We further found that perceived formidability was predicted by the F_0_, HNR, intensity, and duration. For full detail, see Table 4.

**Table 4:** Summary of linear mixed effects model analysis for acoustic predictors of perceived formidability based on utterances and roars.

	**Predictors**	**Estimate**	***t***	***P***	**95% CI**
Utterances	F_0_ (Hz)	−0.017	−3.326	0.002	−0.027, −0.007
	F_3_ (Hz)	−0.0001	−1.213	0.233	−0.003, 0.00006
	HNR (dB)	0.007	0.194	0.847	−0.061, 0.074
	Intensity (dB)	0.140	4.663	<0.001	0.081, 0.199
	Duration (s)	−0.023	−0.691	0.494	−0.088, 0.042
Roars	F_0_ (Hz)	0.005	3.31	0.002	0.002, 0.008
	F_3_ (Hz)	−0.00004	−1.59	0.121	−0.00009, 0.00001
	HNR (dB)	−0.070	−6.13	< 0.001	−0.093, −0.048
	Intensity (dB)	0.911	4.29	< 0.001	0.495, 1.328
	Duration (s)	0.125	6.96	< 0.001	0.090, 0.160

### Acoustic Predictors of Fighting Success

To explore whether any acoustic parameters predict actual MMA fighting success, we ran a multiple linear regression analysis for both utterances and roars. Overall models were not statistically significant in either utterances or roars [Utterances: *F*_(5, 23)_ = 0.774, *p* = 0.578, R^2^ = 0.144; Roars: *F*_(5, 23)_ = 1.107, *p* = 0.384, R^2^ = 0.194]. For full results, see Table 5.

**Table 5:** Summary of multiple linear regression analysis for acoustic predictors of fighting success based on utterances and roars.

	**Predictors**	**β**	***t***	***p***	**95% CI**
Utterances	F_0_ (Hz)	0.107	0.469	0.644	−0.366, 0.581
	F_3_ (Hz)	−0.076	−0.382	0.706	−0.488, 0.336
	HNR (dB)	−0.036	0.155	0.878	−0.45, 0.522
	Intensity (dB)	0.045	0.181	0.858	−0.469, 0.559
	Duration (s)	−0.338	−1.488	0.150	−0.807, 0.132
Roars	F_0_ (Hz)	0.220	1.476	0.154	−0.185, 1.108
	F_3_ (Hz)	−0.073	−0.318	0.753	−0.472, 0.346
	HNR (dB)	−0.050	−0.093	0.927	−0.420, 0.384
	Intensity (dB)	−0.186	−1.077	0.293	−0.882, 0.278
	Duration (s)	−0.339	−2.023	0.055	−0.921, 0.01

## Discussion

The main goal of this study was to test whether a perception of formidability based on intimidating roars and non-intimidating utterances is related to body parameters such as body height, weight, and to some relevant aspects of physical fitness, such as strength and lung capacity. We have also tested whether perceived formidability is related to actual fighting success. Finally, we performed an acoustic analysis to investigate which parameters predict perceived formidability and fighting success. In contrast to our predictions, we found that neither body height, weight, or muscle mass predict perceived formidability neither from speech not roars. We also found no significant association between formidability of the roars and utterances and actual fighting success. Finally, our acoustic analysis showed that the intensity (the acoustic analog of loudness) of both speech and roars is the strongest predictor of perceived formidability. In roars, but not in utterances, lower HNR and longer duration predicted perceived formidability. Moreover, while lower voices (lower F_0_) were perceived as more formidable in utterances, the opposite held for the roars.

Our negative findings concerning an association between body height and strength of the roars contrast with results reported in a recent paper by Raine et al. (2018a), where the authors found that the listeners could predict relative body height and handgrip strength from both speech and roars. Such results are further supported by another study which showed a positive association between handgrip strength and perceived strength based on speech (Sell et al., 2010). On the other hand, another two studies found no association between threat potential and perceived fighting ability/dominance from speech (Doll et al., 2014; Han et al., 2017).

There are several possible explanations for such striking differences between our study and results reported in Raine et al. First of all, both Raine et al. (2018a) and Sell et al. (2010) asked participants specifically to assess strength, while our participants rated formidability. Although strength does certainly contribute to overall formidability, there are other important factors which influence it, such as agility or endurance. Moreover, differences in the use of perceptual attributes can, too, affect the association with measures of formidability. Since our main goal was to investigate how people perceive threat potential based on acoustic cues, we used a broader concept of formidability instead of focusing narrowly on the perception of strength. To resolve this issue, future studies should compare ratings of strength and formidability based on acoustic cues and its correlates while employing the same set of stimuli (for results based on the perception of faces and bodies, see Sell et al., 2010).

Secondly, Raine et al. (2018a) in their ratings used an ego-centered approach, i.e., their participants assessed strength relatively to their own strength. We agree that perceivers may be particularly sensitive when it comes to estimating their own chances of winning a confrontation. Nonetheless, several other studies did use absolute ratings, including rating of perception of strength from speech (e.g., Sell et al., 2010), and found positive results. It is possible that even under these conditions, people tend to use the scale relatively to their own prospects. It could also be argued that because our targets were experienced fighters, there should be no difference between the relative and absolute ratings because vast majority of student listeners would rate their formidability as lower than that of MMA fighters in either case. This is supported by a comparison of mean values of handgrip strength between our (Table 1) and Raine et al., 2018a (Supplementary Information, p. 4) study, although this is only a very approximate estimate because these two studies used different types of dynamometer and resulting values therefore cannot be directly compared. Alternatively, people might be able to assess formidability irrespective of ego involvement. This is supported by a study which explicitly used the bystander paradigm (Little et al., 2015). In particular, raters were asked to judge from facial photographs who will win a fight and they were successful above the chance level. Once again, to obtain more fine-grained insights into how ego-related context affects the cognitive processes of formidability assessments, future investigations should compare this directly.

Thirdly, in our study we used vocal stimuli from MMA fighters who have extensive experience with physical encounters and some fighters produce roars when winning a fight. It would seem advantageous to employ such a group of participants rather than, for instance, students who are likely to have limited experience with both fighting and roaring. Potential drawback of our sample of fighters may be that because of intense training, they will display little variability in their handgrip strength. Inspection of variation estimates, such as SD, shows that this was not the case (see Table 1). The sample size of our stimuli was rather moderate (*N* = 40), but a related study by Raine et al. (2018a) reported positive effect based on smaller sample of the male stimuli.

Finally, one could argue that formidability perception of the roars is related to the effort. This is supported by our acoustic analysis which showed that intensity and duration was the strongest predictor of formidability judgements. It is thus possible that in our sample, motivation and consequently also effort invested in the roars varied among our participants and as a result may have obscured some of the associations with physical characteristics. Alternatively, and perhaps most importantly, the full expression of intimidating roars is not under complete volitional control, which is why it is possible that it can be expressed only in the appropriate context (e.g., when conflict is imminent). Using on-demand roars might not be a problem for judgements of strength but could be a key factor in formidability inferences. Although we acknowledge that this might be a logistically challenging task, the use of real-life non-verbal vocal stimuli which vary little in their motivation and/or effort should thus be preferred. An excellent example in this context is the study by Raine et al. (2017) who used as their stimuli the grunts of professional tennis players.

The acoustic analysis showed that for formidability judgements, intensity and duration are the most salient predictor. This is in agreement with studies on various vertebrate species. For example, male green frogs (*Rana clamitans*) react differently to calls produced by large males as opposed to small ones (Bee et al., 1999; but see Bee, 2002). Similarly, more dominant male baboons produce longer and louder calls (so called “wahoos”) during contest vocalizations (Fischer et al., 2002; Kitchen et al., 2003). Interestingly, many studies on speech perception standardize their vocal stimuli for intensity (because reliable measures of acoustic intensity are logistically difficult to acquire) and therefore cannot assess intensity's contribution to the respective perceptual attribute. However, our results, as well studies on perception of affective states and intentions (Scherer, 1986, 2003; Siegman et al., 1990; Banse and Scherer, 1996), show that loudness (i.e., the perceptual analog of voice intensity) is an integral and significant part of voice perception. Indeed, the same verbal content expressed in a soft, moderate, or loud voice often has a very different impact on perceivers (Patel et al., 2011). We further found that a low HNR of roars, but not of utterances, is associated with high formidability ratings. Previous studies also show a higher noise in threatening calls than in non-threatening vocalizations and a higher perturbation (lower HNR) in anger vocalization in humans (Patel et al., 2011).

Finally, fundamental frequency was negatively associated with formidability of speech, while associating positively with the formidability of roars. The results of formidability judgements from speech are in agreement with other studies which consistently show that male voices with a lower voice pitch (the perceptual analog of fundamental frequency) are perceived as more dominant and attractive (Puts et al., 2006). This could be a consequence of sex dimorphism in the voice pitch (Rendall et al., 2005; Markova et al., 2016). In contrast, our finding of a positive association between fundamental frequency and formidability judgements of roars came at first as a surprise. On the other hand, one could take into account that high-pitched voices might, similarly to intensity, provide cues about the effort and affective state of the producers, whereby those in a state of high arousal would produce higher F_0_ roars. This speculation is supported by studies showing that arousal leads to increase in voice pitch perhaps as a consequence of tension in glottal area (Ekman et al., 1976; van Mersbergen et al., 2017). Moreover, high pitch is in some species associated with threat vocalizations (Stirling, 1971; Portfors, 2007) and in humans, it is associated with anger vocalizations (Scherer and Oshinsky, 1977; Frick, 1986). Fitch et al. (2002) have proposed that subharmonics (portions of F_0_) in loud calls are more prevalent and one of the hypothesized effects of this phenomenon is that they perceptually lower the pitch. In other words, a loud vocalization of the same individual that has the same F_0_ could sound lower-pitched than if the same vocalization were produced in moderate loudness. Although we were able to detect subharmonics phenomena in a number of high intensity roars in our sample (see Supplementary Material Table S10), this effect should be systematically investigated in future studies.

To summarize, we found no significant association between formidability perception of the intimidating roars produced by the MMA fighters and their body height, weight, and physical fitness indicators such as handgrip strength or lung capacity. Neither did we find a correlation between the perceived formidability of their roars and their actual fighting success. This might be because accurate judgements of formidability can be made only on the basis of real-life roars and cannot be reliably performed on demand. It may also be relevant that while roars might be primarily interpreted as intentions (e.g., as affective state of anger), utterances might be interpreted primarily as characteristic of the individual (e.g., as a level of dominance). Alternatively, the association between some acoustic parameters and perceived formidability might be the result of sensory exploitation and have only limited predictive value for actual formidability (Feinberg et al., 2018). We also found that the main acoustic predictors of formidability in roars are intensity, HNR, duration, and to some extent also fundamental frequency. In a broader context, our study points to a need of further investigations of non-verbal vocalizations in humans. Scholars seem to be so blinded by humans' exceptional gift of speech that they tend to almost completely overlook the fact that this is not our only vocalization. Non-verbal vocalizations are cross-culturally prevalent in human social milieu. This applies not only to preverbal infants (see for instance Lindová et al., 2015) but also to adult humans who produce a wide variety of non-verbal vocalizations in diverse contexts, such as co-laughter, painful injuries, aggressive confrontations, and sexual encounters, to name just few (for some pioneering studies, see Bryant et al., 2016; Raine et al., 2018a,b). We are confident that research into these non-verbal vocal displays will greatly contribute to our understanding of the complexity of human vocal expressions and perhaps also to the evolutionary history of verbal communication in general (Hauser et al., 2002).

## Author Contributions

PŠ, VT, JF, and JH developed the study concept. Data collection was performed by PŠ, VT, and JF. PŠ performed acoustic analysis of vocal stimuli. VT and PŠ performed data analysis and interpretation jointly with JF and JH. JH, PŠ, and VT drafted the manuscript and JF provided critical revisions. All authors approved the final version of the manuscript for submission.

### Conflict of Interest Statement

The authors declare that the research was conducted in the absence of any commercial or financial relationships that could be construed as a potential conflict of interest.

## Supplementary Material

Audio S1 Sample of highly formidable roar.

Audio S2 Sample of low formidable roar.

Audio S3 Sample of highly formidable utterance.

Audio S4 Sample of low formidable utterance.

Figure S1 Sample of failed roar F_0_ measurement spectrogram.

Figure S2 Sample of successful roar FFT spectral peak frequency.

Table S1–S19 Supplementary results.

Table S20 Dataset ratings.

Table S21 Dataset targets.

Table S22 Exploratory correlation table.
