# Supplementary figures and images for: Roar of a Champion: Loudness and Voice Pitch Predict Perceived Fighting Ability but Not Success in MMA Fighters
Source: Front Psychol. 2019 Apr 30;10:859. doi: 10.3389/fpsyg.2019.00859 (PMC6502904; doi:10.3389/fpsyg.2019.00859)

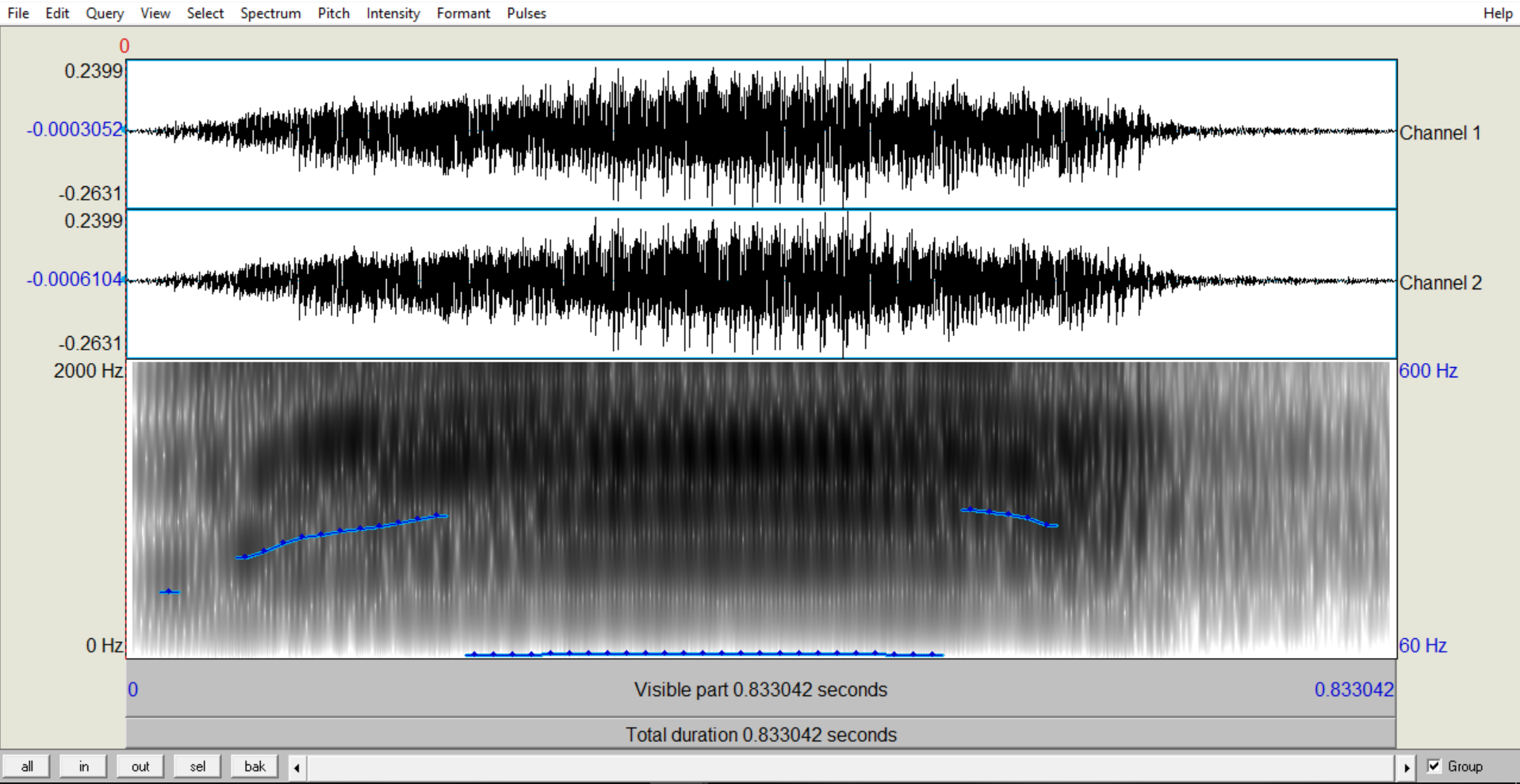

Supplement: Figure S1 — Sample of failed roar F0 measurement spectrogram. [file Image_1.png]

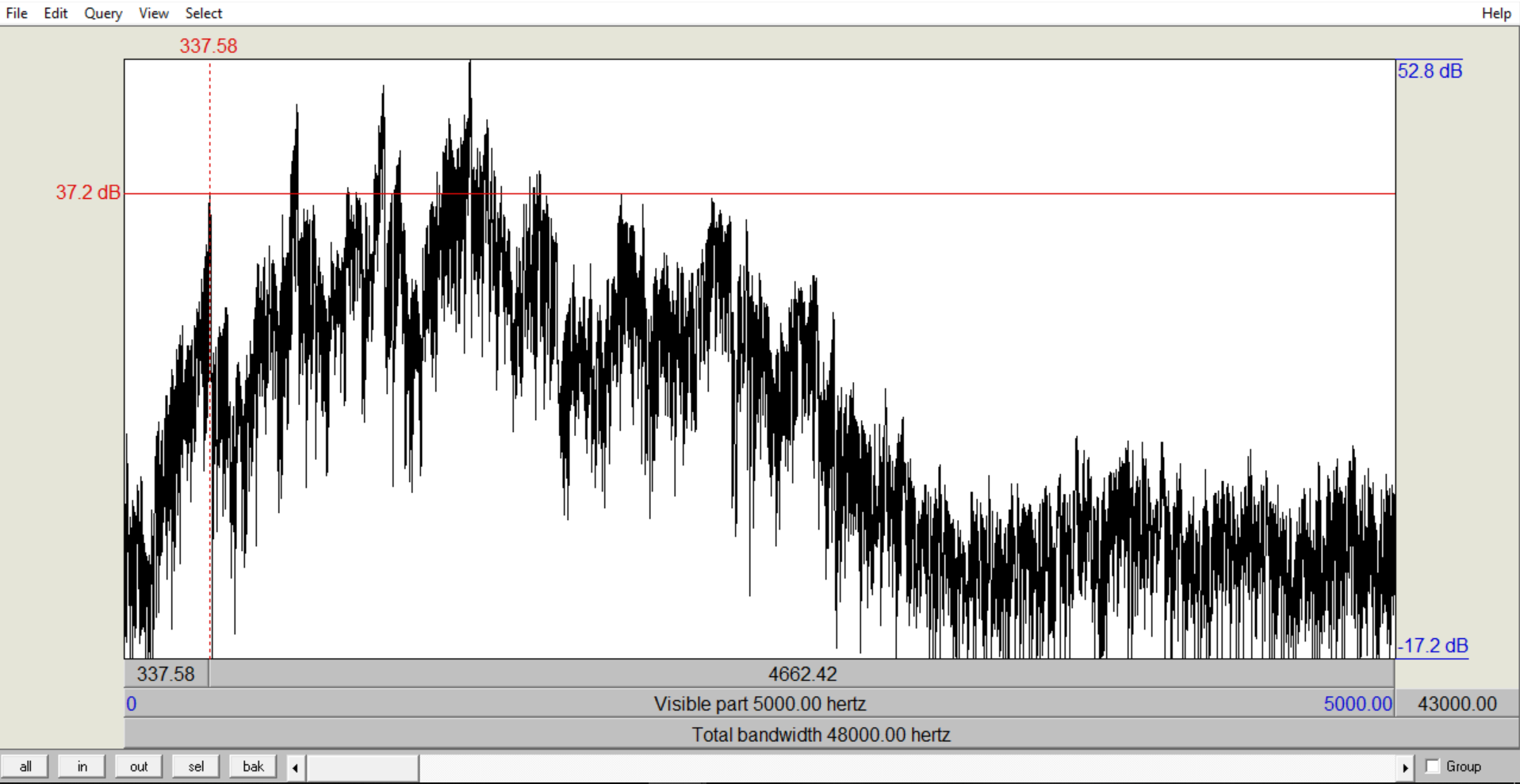

Supplement: Figure S2 — Sample of successful roar FFT spectral peak frequency. [file Image_2.png]
