# Supplementary material for: Roar of a Champion: Loudness and Voice Pitch Predict Perceived Fighting Ability but Not Success in MMA Fighters
Source: Front Psychol. 2019 Apr 30;10:859. doi: 10.3389/fpsyg.2019.00859 (PMC6502904; doi:10.3389/fpsyg.2019.00859)
Supplement: Table S1–S19 — Supplementary results. [file Table_1.pdf]

Supplementary Material

Roar of a Champion: Loudness and Voice Pitch Predict Perceived Fighting Ability But Not Success in MMA Fighters

Pavel Šebesta, Vít Třebický, Jitka Fialová & Jan Havlíček*

*** Correspondence:** jhavlicek@natur.cuni.cz

Table S1 | Descriptive statistics of Roars' acoustic parameters

	Attempt	Mean	SD	Minimum	Maximum
F0 (Hz)	1st roar	385.913	61.397	224.377	492.632
	2nd roar	394.978	55.812	243.503	492.725
	3rd roar	392.557	53.986	214.188	491.509
F3 (Hz)	1st roar	2546.047	220.79	2194	3160
	2nd roar	2621.953	246.95	2196	3150
	3rd roar	2612.93	263.513	2168	3157
HNR (dB)	1st roar	7.886	3.642	1.853	15.913
	2nd roar	7.564	4.26	0.901	19.532
	3rd roar	8.247	5.334	1.54	27.299
Intensity (dB)	1st roar	53.505	5.285	41.169	62.878
	2nd roar	54.73	4.639	44.619	61.819
	3rd roar	54.935	5.141	41.744	64.565
Duration (s)	1st roar	0.638	0.312	0.149	1.491
	2nd roar	0.652	0.308	0.138	1.28
	3rd roar	0.637	0.306	0.117	1.337

Table S2 | Repeated Measures ANOVA for Roars' F_0 (Hz)

Within Subjects Effects

	Sphericity Correction	Sum of Squares	df	Mean Square	F	p	η²
Roar F0	None	1894.676	2	947.338	2.69	0.074	0.06
	Greenhouse-Geisser	1894.676	1.556	1217.858	2.69	0.088	0.06
Residual	None	29581.193	84	352.157			
	Greenhouse-Geisser	29581.193	65.341	452.718			

Between Subjects Effects

	Sum of Squares	df	Mean Square	F	p	η²
Residual	381981.236	42	9094.791			

Tests of Sphericity

	Mauchly's W	p	Greenhouse-Geisser ε	Huynh-Feldt ε
Roar F0	0.714	0.001	0.778	0.802

Note. Type 3 Sums of Squares

Table S3 | Repeated Measures ANOVA for Roars' F_3 (Hz)

Within Subjects Effects

	Sphericity Correction	Sum of Squares	df	Mean Square	F	p	η²
Roar F3	None	147872.992	2	73936.496	3.861	0.025	0.084
	Greenhouse-Geisser	147872.992	1.935	76421.127	3.861	0.026	0.084
Residual	None	1.61E+06	84	19149.036			
	Greenhouse-Geisser	1.61E+06	81.269	19792.538			

Between Subjects Effects

	Sum of Squares	df	Mean Square	F	p	η²
Residual	5.92E+06	42	140873.467			

Tests of Sphericity

	Mauchly's W	p	Greenhouse-Geisser ε	Huynh-Feldt ε
Roar F3	0.966	0.496	0.967	1

Post Hoc Comparisons - Roar F3

Comparison							
Roar F3		Roar F3	Mean Difference	SE	df	t	ptukey
1st roar	-	2nd roar	-75.907	29.844	84	-2.543	0.034
	-	3rd roar	-66.884	29.844	84	-2.241	0.07
2nd roar	-	3rd roar	9.023	29.844	84	0.302	0.951

Note. Type 3 Sums of Squares

Table S4 | Repeated Measures ANOVA for Roars' HNR (dB)

Within Subjects Effects

	Sphericity Correction	Sum of Squares	df	Mean Square	F	p	η²
Roar HNR	None	10.04	2	5.021	0.685	0.507	0.016
	Greenhouse-Geisser	10.04	1.379	7.28	0.685	0.456	0.016
Residual	None	615.5	84	7.328			
	Greenhouse-Geisser	615.5	57.94	10.62			

Between Subjects Effects

	Sum of Squares	df	Mean Square	F	p	η²
Residual	1899	42	45.22			

Tests of Sphericity

	Mauchly's W	p	Greenhouse-Geisser ε	Huynh-Feldt ε
Roar HNR	0.55	< 0.001	0.69	0.706

Note. Type 3 Sums of Squares

Table S5 | Repeated Measures ANOVA for Roars' intensity (dB)

Within Subjects Effects

	Sphericity Correction	Sum of Squares	df	Mean Square	F	p	η²
Roar intensity	None	50.228	2	25.114	8.971	< 0.001	0.18
	Greenhouse-Geisser	50.228	1.746	28.776	8.971	< 0.001	0.18
Residual	None	229.562	82	2.8			
	Greenhouse-Geisser	229.562	71.566	3.208			

Between Subjects Effects

	Sum of Squares	df	Mean Square	F	p	η²
Residual	2881.603	41	70.283			

Tests of Sphericity

	Mauchly's W	p	Greenhouse-Geisser ε	Huynh-Feldt ε
Roar intensity	0.854	0.043	0.873	0.908

Post Hoc Comparisons - Roar intensity

Comparison							
**Roar intensity**		**Roar intensity**	**Mean Difference**	**SE**	**df**	**t**	**ptukey**
1st roar	-	2nd roar	-1.225	0.365	82	-3.354	0.003
	-	3rd roar	-1.43	0.365	82	-3.917	< 0.001
2nd roar	-	3rd roar	-0.206	0.365	82	-0.563	0.84

Note. Type 3 Sums of Squares

Table S6 | Repeated Measures ANOVA for Roars' duration (s)

Within Subjects Effects

	Sphericity Correction	Sum of Squares	df	Mean Square	F	p	η²
Roar duration	None	0.006	2	0.003	0.379	0.686	0.009
	Greenhouse-Geisser	0.006	1.633	0.003	0.379	0.643	0.009
Residual	None	0.606	82	0.007			
	Greenhouse-Geisser	0.606	66.942	0.009			

Between Subjects Effects

	Sum of Squares	df	Mean Square	F	p	η²
Residual	11.107	41	0.271			

Tests of Sphericity

	Mauchly's W	p	Greenhouse-Geisser ε	Huynh-Feldt ε
Roar duration	0.775	0.006	0.816	0.846

Note. Type 3 Sums of Squares

Table S7 | Correlations of F0 between individual Roar attempts

	Statistics	1st roar F0 (Hz)	2nd roar F0 (Hz)		3rd roar F0 (Hz)	
1st roar F0 (Hz)	Pearson's r	—	**0.926**	***	**0.845**	***
	p-value	—	< 0.001		< 0.001	
	95% CI Upper	—	0.959		0.914	
	95% CI Lower	—	0.866		0.731	
2nd roar F0 (Hz)	Pearson's r		—		**0.919**	***
	p-value		—		< 0.001	
	95% CI Upper		—		0.955	
	95% CI Lower		—		0.854	
3rd roar F0 (Hz)	Pearson's r				—	
	p-value				—	
	95% CI Upper				—	
	95% CI Lower				—	

Note. * $p < .05$, ** $p < .01$, *** $p < .001$

Table S8 | Correlations of F3 between individual Roar attempts

	Statistics	1st roar F3 (Hz)	2nd roar F3 (Hz)		3rd roar F3 (Hz)	
1st roar F3 (Hz)	Pearson's r	—	**0.719**	***	**0.648**	***
	p-value	—	< 0.001		< 0.001	
	95% CI Upper	—	0.838		0.794	
	95% CI Lower	—	0.533		0.432	
2nd roar F3 (Hz)	Pearson's r		—		**0.689**	***
	p-value		—		< 0.001	
	95% CI Upper		—		0.819	
	95% CI Lower		—		0.49	
3rd roar F3 (Hz)	Pearson's r				—	
	p-value				—	
	95% CI Upper				—	
	95% CI Lower				—	

Note. * $p < .05$, ** $p < .01$, *** $p < .001$

Table S9 | Correlations of HNR between individual Roar attempts

	Statistics	1st roar HNR (dB)	2nd roar HNR (dB)		3rd roar HNR (dB)	
1st roar HNR (dB)	Pearson's r	—	**0.656**	***	**0.446**	**
	p-value	—	< 0.001		0.003	
	95% CI Upper	—	0.799		0.658	
	95% CI Lower	—	0.443		0.169	
2nd roar HNR (dB)	Pearson's r		—		**0.837**	***
	p-value		—		< 0.001	
	95% CI Upper		—		0.909	
	95% CI Lower		—		0.718	
3rd roar HNR (dB)	Pearson's r				—	
	p-value				—	
	95% CI Upper				—	
	95% CI Lower				—	

Note. * $p < .05$, ** $p < .01$, *** $p < .001$

Table S10 | Correlations of intensity between individual Roar attempts

	Statistics	1st roar intensity (dB)	2nd roar intensity (dB)		3rd roar intensity (dB)	
1st roar intensity (dB)	Pearson's r	—	**0.922**	***	**0.858**	***
	p-value	—	< 0.001		< 0.001	
	95% CI Upper	—	0.957		0.922	
	95% CI Lower	—	0.858		0.75	
2nd roar intensity (dB)	Pearson's r		—		**0.904**	***
	p-value		—		< 0.001	
	95% CI Upper		—		0.948	
	95% CI Lower		—		0.827	
3rd roar intensity (dB)	Pearson's r				—	
	p-value				—	
	95% CI Upper				—	
	95% CI Lower				—	

Note. * p < .05, ** p < .01, *** p < .001

Table S11 | Correlations of duration between individual Roar attempts

	Statistics	1st roar duration (s)	2nd roar duration (s)		3rd roar duration (s)	
1st roar duration (s)	Pearson's r	—	**0.938**	***	**0.886**	***
	p-value	—	< 0.001		< 0.001	
	95% CI Upper	—	0.966		0.937	
	95% CI Lower	—	0.887		0.797	
2nd roar duration (s)	Pearson's r		—		**0.943**	***
	p-value		—		< 0.001	
	95% CI Upper		—		0.969	
	95% CI Lower		—		0.896	
3rd roar duration (s)	Pearson's r				—	
	p-value				—	
	95% CI Upper				—	
	95% CI Lower				—	

Note. * p < .05, ** p < .01, *** p < .001

Table S12 | Linear mixed effect model analysis for physical fitness predictors of perceived fighting ability from roars based on female and male ratings

**Info**		
Estimate	Linear mixed model fit by REML	
Call	Roar_rating ~ 1 + (1 | Rater_id) + (1 | Target_id) + age + height + weight + Handgrip_mean + FVC_l + PEF	
	Female ratings	Male ratings
AIC	3704.6729	3523.5605
R-squared Marginal	0.0926	0.0720
R-squared Conditional	0.5977	0.6227

Table S13 | Summary of linear mixed effect model analysis for physical fitness predictors of perceived fighting ability from roars based on female and male ratings

	Coefficients	Estimate	*t*	p	95% CI
Female ratings	Age (yrs)	0.079	1.536	0.136	-0.022, 0.181
	Height (cm)	-0.045	-0.879	0.387	-0.146, 0.056
	Weight (kg)	0.017	0.889	0.382	-0.020, 0.053
	FVC (l)	0.482	1.692	0.102	-0.077, 1.041
	PEF (l)	-0.130	-1.311	0.201	-0.324, 0.064
	Handgrip strength (kg)	0.003	0.010	0.921	-0.057, 0.063
Male ratings	Age (yrs)	0.067	1.447	0.159	-0.024, 0.159
	Height (cm)	-0.042	-0.907	0.372	-0.132, 0.049
	Weight (kg)	0.018	1.047	0.304	-0.015, 0.051
	FVC (l)	0.322	1.256	0.220	-0.181, 0.825
	PEF (l)	-0.098	-1.106	0.279	-0.273, 0.076
	Handgrip strength (kg)	0.006	0.209	0.836	-0.048, 0.064

Table S14 | Linear mixed effect model analysis for physical fitness predictors of perceived fighting ability from utterances based on female and male ratings

**Info**		
Estimate	Linear mixed model fit by REML	
Call	Utterance_rating ~ 1 + (1 | Rater_id) + (1 | Target_id) + age + height + weight + Handgrip_mean + FVC_l + PEF	
	Female ratings	Male ratings
AIC	3616.6268	3481.6454
R-squared Marginal	0.0536	0.0554
R-squared Conditional	0.4115	0.4750

Table S15 | Summary of linear mixed effect model analysis for physical fitness predictors of perceived fighting ability from utterances based on female and male ratings

	Coefficients	Estimate	*t*	p	95% CI
Female ratings	Age (yrs)	0.019	0.727	0.474	-0.033, 0.071
	Height (cm)	0.047	1.799	0.083	-0.004, 0.099
	Weight (kg)	-0.009	-0.915	0.368	-0.027, 0.010
	FVC (l)	-0.287	-1.971	0.059	-0.573, -0.002
	PEF (l)	0.021	0.411	0.684	-0.078, 0.120
	Handgrip strength (kg)	0.013	0.808	0.426	-0.018, 0.043
Male ratings	Age (yrs)	0.028	1.075	0.292	-0.023, 0.080
	Height (cm)	0.052	1.994	0.056	0.020, 0.103
	Weight (kg)	-0.011	-1.114	0.275	-0.029, 0.008
	FVC (l)	-0.267	-1.841	0.077	-0.552, 0.017
	PEF (l)	0.011	0.219	0.828	-0.088, 0.110
	Handgrip strength (kg)	0.010	0.626	0.537	-0.021, 0.040

Table S16 | Linear mixed effect model analysis for acoustic predictors of perceived fighting ability from roars based on female and male ratings

**Info**		
Estimate	Linear mixed model fit by REML	
Call	Roar_rating ~ 1 + (1 | Rater_id) + (1 | Target_id) + `Shout_F0_(h1)_hz` + `Shout_F3_(khz2)_hz` + Shout_HNR_dB + Shout_duration_s + shout_intensity_mean_dB	
	Female ratings	Male ratings
AIC	4272.859	4107.777
R-squared Marginal	0.413	0.339
R-squared Conditional	0.566	0.579

Table S17 | Summary of linear mixed effect model analysis for acoustic predictors of perceived fighting ability from roars based on female and male ratings

	Coefficients	Estimate	*t*	p	95% CI
Female ratings	F_0 (Hz)	0.006	3.620	< 0.001	0.003, 0.009
	F_3 (Hz)	-0.0002	-0.762	0.451	-0.0007, 0.0003
	HNR (dB)	-0.075	-6.489	< 0.001	-0.098, -0.052
	Intensity (dB)	0.128	7.057	< 0.001	0.092, 0.164
	Duration (s)	1.165	5.420	< 0.001	0.743, 1.586
Male ratings	F_0 (Hz)	0.005	2.72	0.010	0.001, 0.008
	F_3 (Hz)	-0.00006	-2.22	0.033	-0.001, -0.00001
	HNR (dB)	-0.065	-5.25	< 0.001	-0.090, -0.041
	Intensity (dB)	0.122	6.23	< 0.001	0.083, 0.160
	Duration (s)	0.658	2.85	0.007	0.205, 1.111

Table S18 | Linear mixed effect model analysis for acoustic predictors of perceived fighting ability from utterances based on female and male ratings

**Info**		
Estimate	Linear mixed model fit by REML	
Call	Utterance_rating ~ 1 + (1 | Rater_id) + (1 | Target_id) + ` Utterance _F0_(h1)_hz` + ` Utterance _F3_(khz2)_hz` + Utterance _HNR_dB + Utterance _duration_s + Utterance _intensity_mean_dB	
	Female ratings	Male ratings
AIC	4260.9175	4094.7579
R-squared Marginal	0.0993	0.0954
R-squared Conditional	0.4026	0.4653

Table S19 | Summary of linear mixed effect model analysis for acoustic predictors of perceived fighting ability from utterances based on female and male ratings

	Coefficients	Estimate	*t*	p	95% CI
Female ratings	F_0 (Hz)	-0.016	-3.028	0.005	-0.026, -0.006
	F_3 (Hz)	−0.0001	-1.147	0.260	-0.003, 0.00007
	HNR (dB)	0.013	0.357	0.723	-0.058, 0.084
	Intensity (dB)	0.143	4.503	< 0.001	0.080, 0.205
	Duration (s)	-0.025	-0.727	0.472	-0.094, 0.043
Male ratings	F_0 (Hz)	-0.018	-3.402	0.002	-0.028, -0.007
	F_3 (Hz)	-0.001	-1.198	0.239	-0.003, 0.0006
	HNR (dB)	0.00004	0.013	0.990	-0.068, 0.069
	Intensity (dB)	0.138	4.505	< 0.001	0.078, 0.198
	Duration (s)	-0.021	-0.606	0.548	-0.087, 0.046
